# A novel allogeneic off-the-shelf dendritic cell vaccine for post-remission treatment of elderly patients with acute myeloid leukemia

**DOI:** 10.1007/s00262-018-2198-9

**Published:** 2018-07-23

**Authors:** Arjan A. van de Loosdrecht, Sandra van Wetering, Saskia J. A. M. Santegoets, Satwinder Kaur Singh, Corien M. Eeltink, Yvonne den Hartog, Malika Koppes, Jorn Kaspers, Gert J. Ossenkoppele, Ada M. Kruisbeek, Tanja D. de Gruijl

**Affiliations:** 1 Department of Hematology, VU University Medical Center, Cancer Center Amsterdam, De Boelelaan 1117, 1081 HV Amsterdam, The Netherlands; 2 DCPrime BV, Galileiweg 8, 2333 BD Leiden, The Netherlands; 3 Department of Medical Oncology, VU University Medical Center, Cancer Center Amsterdam, De Boelelaan 1117, 1081 HV Amsterdam, The Netherlands; 4 Department of Medical Oncology, Leiden University Medical Center, Leiden, The Netherlands

**Keywords:** Dendritic cells, Immune therapy, Acute myeloid leukemia, Phase I trial

## Abstract

In elderly acute myeloid leukemia (AML) patients post-remission treatment options are associated with high comorbidity rates and poor survival. Dendritic cell (DC)-based immunotherapy is a promising alternative treatment strategy. A novel allogeneic DC vaccine, DCP-001, was developed from an AML-derived cell line that uniquely combines the positive features of allogeneic DC vaccines and expression of multi-leukemia-associated antigens. Here, we present data from a phase I study conducted with DCP-001 in 12 advanced-stage elderly AML patients. Patients enrolled were in complete remission (CR1/CR2) (*n* = 5) or had smoldering disease (*n* = 7). All patients were at high risk of relapse and ineligible for post-remission intensification therapies. A standard 3 + 3 dose escalation design with extension to six patients in the highest dose was performed. Patients received four biweekly intradermal DCP-001 injections at different dose levels (10, 25, and 50 million cells DCP-001) and were monitored for clinical and immunological responses. Primary objectives of the study (feasibility and safety) were achieved with 10/12 patients completing the vaccination program. Treatment was well tolerated. A clear-cut distinction between patients with and without detectable circulating leukemic blasts during the vaccination period was noted. Patients with no circulating blasts showed an unusually prolonged survival [median overall survival 36 months (range 7–63) from the start of vaccination] whereas patients with circulating blasts, died within 6 months. Long-term survival was correlated with maintained T cell levels and induction of multi-functional immune responses. It is concluded that DCP-001 in elderly AML patients is safe, feasible and generates both cellular and humoral immune responses.

**Electronic supplementary material:**

The online version of this article (10.1007/s00262-018-2198-9) contains supplementary material, which is available to authorized users.

## Introduction

Acute myeloid leukemia (AML) most commonly affects the elderly population with a median age of around 67 years [1, 2]. Current AML treatment relies largely on intensive chemotherapy and allogeneic hematopoietic stem cell transplantation (HSCT), which is unsuccessful in 60–80% of patients due to persistence of measurable residual disease (MRD) [3, 4]. Particularly in elderly patients, these strategies are associated with high comorbidity rates and the 5-year overall survival in this population remains poor (10–15% in patients > 65). Therefore, new treatment strategies are urgently needed.

Immunotherapeutic approaches aiming to eradicate MRD through activation of CD8^+^ cytotoxic T-lymphocytes (CTL), contribute to improved disease outcome [5]. Such anti-leukemia CTL are most effectively primed by the most powerful antigen-presenting cell identified to date, i.e., the dendritic cell (DC). Clinical trials have shown that DC vaccination is safe, has hardly any side effects and induces immune responses [6, 7]. However, autologous DC vaccination is cumbersome, costly and logistically complex. Moreover, clinical efficacy has proven limited, most probably due to the fact that most trials were performed with single-antigen-loaded DC. As an alternative, the use of autologous or allogeneic whole tumor cell lysates or tumor-derived peptide pools as a source of tumor-associated antigens (TAAs) for DC loading has been extensively explored [8, 9]. These formulations cover a wide range of both uncharacterized and characterized tumor antigens, thereby reducing the chance of post-vaccination immune escape. Unfortunately, feasibility was often limited by availability of sufficient numbers of autologous or allogeneic tumor cells [10].

Allogeneic DCs have been reported to induce stronger antigen-specific immune responses than autologous DC because they trigger a broader CD8^+^ T cell immune repertoire, including tumor reactive T cells, and a broad inflammatory response by polyclonal stimulation of allogeneic T cells [11, 12]. The indirect antigen presentation route by host DCs and strong Th1 cell differentiation and activation in response to allo-antigens, will add to the T cell-mediated antitumor response.

A novel DC vaccine, DCP-001, was developed that uniquely combines the positive features of allogeneic DC vaccines and multi-antigen-expressing tumor cell vaccines. DCP-001 consists of mature DC generated through differentiation and maturation of the AML cell line DCOne and as such harbors AML-associated antigens.

Here, we report the results of a phase-I safety and feasibility trial of DCP-001 vaccination in advanced-stage elderly AML patients ineligible for standard post-remission therapies. Besides clinical safety, the results demonstrate biological efficacy of the vaccine with the induction of both specific T cell and humoral responses. Moreover, clinical efficacy is suggested by an unexpectedly sustainable clinical benefit observed in five out of the twelve enrolled patients.

## Materials and methods

### Study design and patient inclusion

A phase I feasibility and safety trial was conducted from March 2011 to March 2013. The set-up followed a standard 3 + 3 dose escalation design with extension to six patients in the highest non-toxic dose level. Main inclusion criteria were either AML in second complete remission (CR2), (relapsed) smoldering AML, or de novo AML in CR1, all not eligible for additional intensification therapy. Patient characteristics are listed in Table 1. Exclusion criteria included uncontrolled active infection, previous immunotherapy in the last 3 months, and previous allogeneic peripheral stem cell transplantation. Patients received DCP-001 vaccinations at days 0, 14, 28 and 42. Skin testing was performed at days 2, 49 and 51. For immunomonitoring, samples were taken before first vaccination, 1 week after the fourth vaccination (day 49), and 12 weeks after the fourth vaccination (day 126). Subsequently, extended follow-up until death was performed to evaluate clinical outcome.

**Table 1:** Patient characteristics

Patient no.	Age	Sex	Time between AML diagnosis and study entry (Mo)	Disease status		Dead/alive at end of study	% Blasts in bone marrow (cytomorphology)	
				At study entry	At end of study		At study entry	At end of study
DC-001	66	F	8	AML relapse/smoldering	CR	Alive	7	3
DC-002	70	M	7	AML relapse/smoldering	Disease progression	Dead	53	89
DC-004	72	F	45	AML in CR2	CR	Alive	2	3
DC-005	74	M	75	AML relapse/smoldering	Pneumonia	Dead	58	MD^a^
DC-006	69	F	18	AML relapse/smoldering	Disease progression	Alive	14	80
DC-007	74	F	14	AML relapse/smoldering	Smoldering disease	Alive	MD	7
DC-008	64	F	7	de novo AML, in CR1	CR	Alive	0	MD
DC-011	57	M	11	de novo AML, in CR1	Endocarditis	Dead	1	MD^a^
DC-012	70	M	22	AML relapse/smoldering	Disease progression	Alive	5	31
DC-013	65	M	3	de novo AML, in CR1	CR	Alive	2	2
DC-014	67	M	20	AML relapse/smoldering	Disease progression	Dead	29	MD^a^
DC-015	68	M	5	de novo AML, in CR1	CR	Alive	7	1

*MD* missing data, *Mo* months, *No* number

^a^Died before end of study

The primary endpoints were safety and feasibility, secondary endpoints included evaluation of immune responses.

#### DCP-001 production

**Fig. 1:**
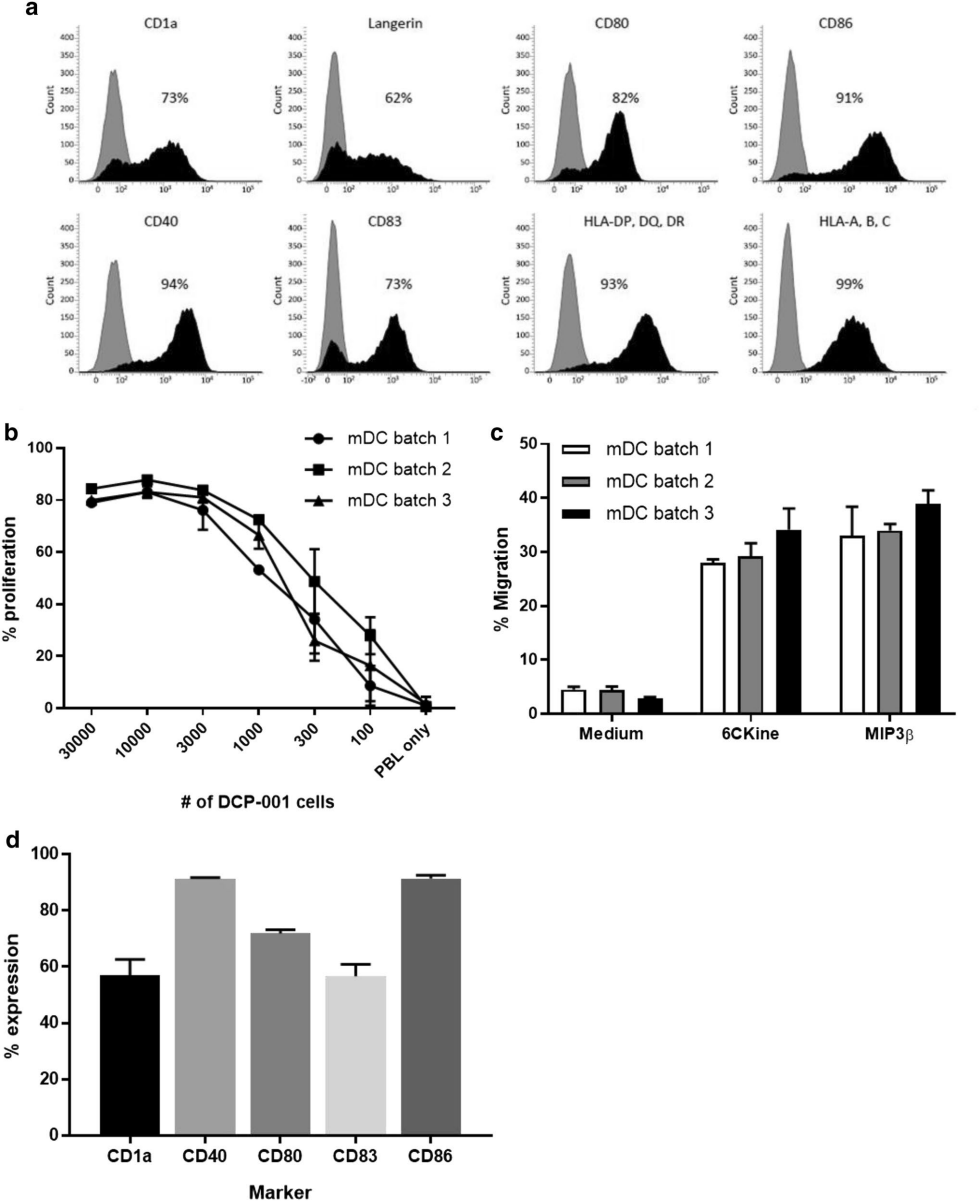
Phenotype, T cell stimulatory and migratory capacity of DCP-001. DCP-001 was analyzed for **a** expression of dendritic cell markers and costimulatory molecules, **b** its allogeneic T cell stimulatory capacity and **c** ability to migrate to lymph node homing chemokines 6CKine and MIP3ß. **a** Expression levels of CD1a, langerin, and several costimulatory molecules were analyzed by flow cytometry; isotype-matched controls (shaded histograms) and the tested markers (closed histograms) are indicated. Mean fluorescence is shown in each panel. **b** Allogeneic T cell stimulatory capacity was analyzed by MLR. Proliferation of CFSE-labeled PBL was assessed after culture for 6 days with a dose range of DCP-001 cells as stimulators. CFSE dilution was used as a measure of percentage of proliferated cells. Results of three different DCP-001 batches, each as mean ± SD performed in six replicates. **c** Analysis of migratory capacity of DCP-001. Cells were analyzed for their capacity to migrate toward LN homing chemokines in a trans-well migration assay. Migration toward medium, 6CKine and MIP3ß is given as a percentage of migrated cells. Data represent mean ± SD of three independent batches of DCP-001 each performed in triplicate. Each batch refers to a clinical batch. **d** QC release on phenotype for DCP-001 batches. Results show the mean ± SD of three independently produced clinical batches. The %CV between the batches is < 10% pointing to a high-batch comparability

DCP-001 was manufactured according to good manufacturing practice (GMP) regulations following previously described protocols [13, 14]. In brief, the cells were cultured in a cocktail of GM-CSF, TNFα, and IL-4 in the presence of mitoxantrone to accelerate DC differentiation, followed by maturation in the presence of prostaglandin-E2, TNFα, and IL-1β. Quality control (QC) included microbiological controls and QC release tests for cell viability and number, phenotype (by flow cytometry, based on expression of CD1a, Langerin, and MHC and costimulatory markers), and potency through T cell priming in a mixed leukocyte reaction (MLR), and migration in response to the lymph node homing chemokines MIP3β and 6-CKine in a trans-well assay—all as described earlier [13, 15–17]. In total, three different GMP batches were prepared and shown to be highly comparable in terms of phenotype and functionality (Fig. 1). Clinical lots were gamma irradiated to prevent cell replication and cryopreserved in Cryostor CS10 (Biolife Solutions, USA).

#### DCP-001 administration

Patients received four biweekly intradermal (i.d.) DCP-001 vaccinations (2–4 four injections of 0.5 mL each) in the upper leg. The first cohort (*n* = 3; patient 001, 002, 004) received 10 million cells/vaccination, the second cohort (*n* = 3; patient 005, 006, 007) 25 million cells/vaccination, and the third cohort (*n* = 3 patient 008, 011, 012) received 50 million cells/vaccination. The third cohort was extended with three patients (patient 013, 014, 015). Delayed-type hypersensitivity reactions were measured in the lower arm. Of note, patient 001 received two booster vaccinations of 10 million cells, 13.5 months after the fourth vaccination (*t* = 0) at 2-week intervals. At this point as well as 21 and 70 days later, blood was drawn for immunomonitoring.

### Clinical sample handling and storage

Peripheral blood mononuclear cells (PBMC) were isolated by Ficoll density separation [16] at day 0, day 49 (1 week after fourth vaccination) and day 126 (12 weeks after fourth vaccination). Serum was collected at similar time points and stored at − 80 °C. Monocytes and peripheral blood lymphocytes (PBL) were isolated from PBMC by CD14 magnetic bead isolation (MACS, Miltenyi Biotec, Germany) following manufacturers’ instructions. PBMC, PBL and monocytes were cryopreserved in C.T.L. CryoABC cryopreservation medium (ImmunoSpot, Cleveland, USA).

### HLA typing

At screening, all patients were serologically typed for HLA class I and II by Sanquin, Amsterdam, Netherlands.

### Delayed-type hypersensitivity (DTH) testing

DTH testing was performed to monitor patient’s cell-mediated immunity before and after treatment by i.d. injection of DCP-001 (2 million cells/0.2 mL) or vehicle control (CS10), at start of vaccination and 7 days after the fourth vaccination. Induration was measured in mm across two diameters after 48 h. Results were expressed as the mean induration. Positive DTH reactions were defined as > 5 mm diameter induration. A difference between first (Pre-Vacc) and second (Post-Vacc) skin test reactions exceeding 50% of the Pre-Vacc measurement, with a Post-Vacc reactivity > 5 mm, was considered to reflect positive vaccination reactivity.

### Immunohistochemistry of DTH site biopsies

Immunohistochemistry analysis was performed on biopsies taken from the DTH sites under local anesthesia. Biopsies were formalin-fixed/paraffin-embedded and stained with the following antibodies as previously described [18–21]: CD1a, CD3, CD4, CD8, CD83, DC-SIGN, Granzyme B, FoxP3, CD45RO, TIA, CD56 and langerin. Positively stained cell rates were assessed independently by two observers and classified as negative (−), low (±), moderate (+), high (++) or very high-infiltration rate (+++). In case of inconsistent results between the two observers, assessment by a third observer was carried out and subsequent consensus reached.

### T cell proliferation and cytokine analysis

Isolated PBL were thawed, CFSE labeled (2 µM) and co-cultured for 7–10 days with different doses of irradiated (50 Gy) monocytes, DCP-001 and DCOne progenitor cells. Cells were stained with fluorochrome-labeled anti-CD3 and anti-CD8 antibodies and proliferation of CD4^+^ (i.e., CD8^−^) and CD8^+^ T cells was measured on a FACSCalibur, using Cellquest software.

The threshold for a positive response was arbitrarily set at 20% proliferation of the responder T cells with low (< 5%) proliferation rates against autologous monocytes. A positive vaccination-induced response was defined as an increase in Post-Vacc proliferation of ≥ 10%. Similarly, an MLR was carried out with PBL from one pre-specified healthy donor to qualify DCP-001. For cytokine analysis, supernatant was harvested and used for T cell cytokine analysis using the Th1/Th2/Th17 Cytometric Bead Array (CBA) kit according to the manufacturer’s instructions (BD Biosciences).

### In vitro T cell stimulation and IFNγ ELISpot analysis

DCP-001 induced T cell responses to full-length TAAs WT-1, PRAME, MAGE-A3 or NY-ESO-1 were measured by IFNγ ELISpot assay as previously described [16, 22, 23]. In brief, overlapping 15-mer peptide pools (JPT peptide technologies, Berlin, Germany) were loaded onto autologous irradiated PBMC which were co-cultured with an equal number of non-irradiated PBMC for 10 days. At day 10, the cells were seeded in an ELISpot plate in the presence of the corresponding peptide pools for 24 h after which an ELISpot read-out was performed. A 15-mer CEFT peptide pool served as a positive recall antigen response control. PHA was used for technical and sample quality control. The ELISpot assay was performed using an anti-IFNγ mAb pair (Mabtech, Nacka, Sweden [23]). T cell activity was expressed as the number of spots per 100,000 T cells (determined by CD3 FACS analysis at the time of ELISpot read-out) and considered positive when, (1) the number of spots in the TAA test condition was significantly higher than the number of spots in the HIV control condition (unpaired Student’s *t* test), (2) the mean number of spots of the test condition exceeded the number of spots of the control condition by at least twofold and (3) the absolute difference in number of spots between the test and control condition was at least five.

### Serology

Vaccination-induced antibodies against DCOne progenitor and, if available, autologous blast lysates were measured in serum by western blot analysis using denaturing 7% SDS–polyacrylamide gels and PVDF protein membranes (BioRad). Following blocking, the membranes were incubated with pre- and post-vaccination sera and HRP-conjugated anti-hIgG/A/M as secondary antibody (Dako). Blots were developed with chemoluminescence substrate (GE Healthcare). Increased intensity or appearance of new bands in Post-Vacc samples denoted DCP-001 vaccination-induced antibody responses.

### Statistical analyses

Differences between immune parameters were assessed before and after treatment with two-sided *t* test. T cell response rates (overall T cell scores) between short- and long-term survivors were compared using the Fisher’s exact test. For data collection, Microsoft Excel (version 2007) was used and for statistical analysis GraphPad Prism software (version 5.0). Differences were considered significant when *p* < 0.05.

## Results

### DCP-001: phenotypic and functional specifications

DCP-001 consists of mature DC differentiated from the AML cell line DCOne which expresses multiple TAAs (e.g., WT-1, PRAME, data not shown). In addition, DCP-001 cells display high expression levels of CD1a, langerin, a wide range of costimulatory molecules, and MHC class I/II molecules (Fig. 1a). The manufactured DCP-001 batches passed QC release tests, including sterility and endotoxin level (< 5 EU/mL). Cell number, viability, phenotypic expression and potency (migration, MLR) were evaluated directly after thawing. DCP-001 cells displayed a consistent ability to prime allogeneic T cells (Fig. 1b) and to migrate in response to the chemokines 6Ckine and MIP3β (Fig. 1c), both involved in homing to the paracortical T cell areas of lymph nodes. Figure 1d depicts the phenotype used for QC release and demonstrates a very high consistency between the 3 independently produced clinical batches.

### Patient characteristics and on-treatment disease development

The safety, feasibility, and biological effects of DCP-001 vaccination were investigated in 12 elderly AML patients in a 3 + 3 dose design. Final assessment was done after 126 days, i.e., 3 months after the last (4th) vaccination. Patient characteristics are listed in Table 1. Blast percentages as listed in Table 1 were based on standard cytomorphology. For listing of prior therapies and cytogenetic features of the patient’s tumors, we refer to Supplementary Table 1. Patients (age range 58–71), enrolled were either in CR1/CR2 (*n* = 5) or had smoldering disease (*n* = 7). Twelve patients initiated vaccination of which ten patients received all four vaccinations (83,3%) and two patients (002 and 011) received three vaccinations. One patient discontinued due to disease progression (002) and one patient (011) due to a candida endocarditis.

### Safety and feasibility

**Fig. 2:**
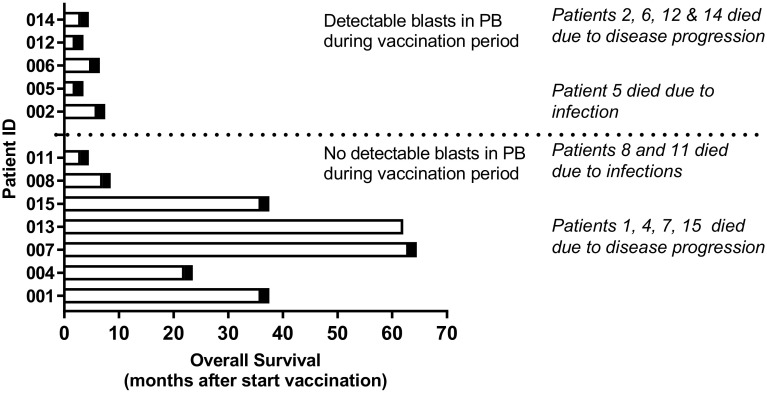
Detectability of leukemic blasts in patients is related to post-vaccination survival. Shown is overall survival since start of vaccination; patients were subdivided by the presence of detectable leukemic blasts in peripheral blood during treatment (dotted line). Death is indicated by black box; causes of death are listed. *PB* peripheral blood

DCP-001 vaccination was well tolerated, safe and feasible. Six patients experienced severe adverse events (SAE) during the study (002, 004, 005, 007, 011 and 014), only one (002) was judged to have a possible relationship to study treatment (diabetes insipidus). This could possibly represent a vaccine-induced autoimmune response. All others were considered unrelated or unlikely to be related to study treatment. Two patients died before completing the study due to pneumonia (005) and disease progression (014). Neither of these deaths were related to study treatment. Two patients discontinued study treatment (see above); SAEs in patients 004 (myocardial infarct) and 007 (vasovagal collapse) did not lead to discontinuation and both patients in fact proved to be long-term survivors (Fig. 2). The distribution of AEs between cohorts was uneven but there was no trend suggesting a dose–response relationship for AE occurrence. AEs were of CTC grade 1 or 2 and most were judged as unrelated to study treatment. The most common AEs were injection site reactions (6 patients), anemia (4 patients), thrombocytopenia (3 patients), fatigue (3 patients), pain in extremity (3 patients), and nausea (3 patients).

### Clinical outcome

At the end of the study (day 126), 9 out of 12 (75%) patients were alive. Eight patients completed all assessments and for those four patients who did not complete the study, reasons were disease progression (002), death due to disease progression (014), pneumonitis/pneumonia (005) and candida endocarditis (011).

Six out of 12 patients were in CR (i.e., undetectable AML blasts in blood and < 5% in bone marrow) at study end and 5/12 experienced persistent disease (Table 1). All patients, except for one, who were in CR at end of study, were in CR1 or CR2 at baseline. All but one of the patients with smoldering disease at study entry, had persistent disease at the end of the study (Table 1). One patient (001) with smoldering disease reached CR at the end of the study. This patient had circulating blast counts of 0.05 × 10^9^/L and bone marrow blasts of 7% at baseline. No clear relationship between clinical outcome and administered DCP-001 dose was apparent.

#### Follow-up

**Fig. 3:**
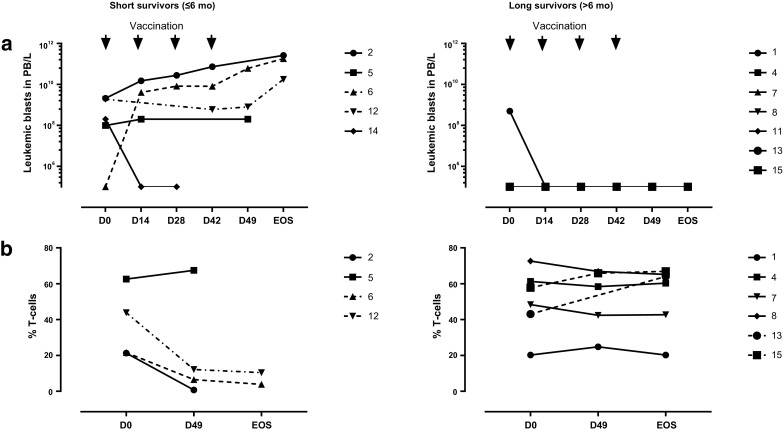
Leukemic blast and T cell rates in short- versus long-term survivors. Absolute numbers of leukemic blasts in peripheral blood over treatment (in days, EOS = end of study) in **a** short-term (less than 6 months) survivors and in **b** long-term (more than 6 months) survivors. T cell rates (as percentage of PBMC) in **c** short-term (less than 6 months) survivors and in **d** long-term (more than 6 months) survivors. *PB* peripheral blood

The study was formally completed in March 2013 but all patients were clinically followed until death. At present (December 2017) one patient is still alive. Based on the presence or absence of circulating leukemic blasts patients could be divided into two groups, which corresponded to short-(≤ 6 months, median overall survival 3 months, range 2–6 months) and long-term survivors (> 6 months, median overall survival 36 months, range 7–63 months), respectively (Fig. 2). The two groups showed strikingly different patterns in peripheral leukemic blast and T cell counts during treatment (Fig. 3). Patients with detectable peripheral blast died within 6 months post-treatment (short-term survivors). Patients without detectable leukemic blasts in peripheral blood (or rapidly dropping below the detection threshold) showed remarkably prolonged survival (Fig. 3a), with one patient still alive at the time of writing and 64 months after study entry and the other patients surviving for 7, 36, 22, 63, and 36 months (Fig. 2). Long-term survival was accompanied by maintained levels of T cells. In patients with short-term survival and detectable circulating blasts, T cell levels dropped precipitously (Fig. 3b).

### Biological efficacy: immune response monitoring

Immune monitoring was performed to evaluate DCP-001 vaccination-induced immune responses and to identify possible relationships to clinical outcome. Patients with detectable blasts at study entry (002, 005, 006, 012, and 014) experienced rapidly dropping T cell frequencies (Fig. 3b) which excluded the possibility to perform all in vitro T cell-based assay in some cases and to draw clear conclusions on immune responses between the two groups. An overview of T cell-related immune data pre- and post-vaccination (day 0, day 49 and day 126) is summarized in Table 3 and are further explained below.

#### HLA-compatibility between DCP-001 and patients

The HLA type of DCP-001 is HLA-A2,3, -B44, -DRB1:10,11 and -DQB1:05‚03. HLA matches varied from 1 to 5 but the number of (mis)matches showed no clear relationship with survival (Table 2). None of the patients showed a full mismatch, i.e., not expressing any of the DCP-001 HLA alleles (Table 2). Moreover, no correlations between degree of HLA match and observed immune responses were found.

**Table 2:** HLA typing of patients

	Patient ID^a^	HLA-A	HLA-B	HLA-C	HLA-DQ	HLA-DR	No. of HLA matches
	DCP-001	A2, 3	B44	C4, 7	DQ3, 5	DR10, 11	
Short survivors ≤ 6 months	002	A1‚ 3	B7‚ 8	C7	DQ2, 6	DR3, 15	2
	005	A1‚ 25	B18, 56	MD	MD	MD	MD
	006	A2, 32	B40, 44	C3, 5	DQ2, 4	DR3, 8	2
	012	A2, 3	B7, 15	C7, 3	DQ3	DR4, 9	3
	014	A1, 3	B15, 39	C3, 5	DQ4, 6	DR8, 13	1
Long survivors > 6 months	001	A3, 24	B15, 44	C3, 5	DQ3, 5	DR4, 14	4
	004	A2	B27, 40	C1, 3	DQ5‚ 3	DR1, 4	2
	007	A2, 31	B15, 40	C3, 3	DQ3, 5	DR4, 16	3
	008	A2, 32	B7, 44	C7, 5	DQ3, 6	DR12, 15	4
	013	A2, 68	B7, 44	C7, 7	DQ3, 6	DR11, 15	5
	015	A3, 11	B44, 55	C3, 3	DQ3, 5	DR4, 16	4

*ID* identity, *MD* missing data, *No* number

#### Antigen-specific immune responses

**Fig. 4:**
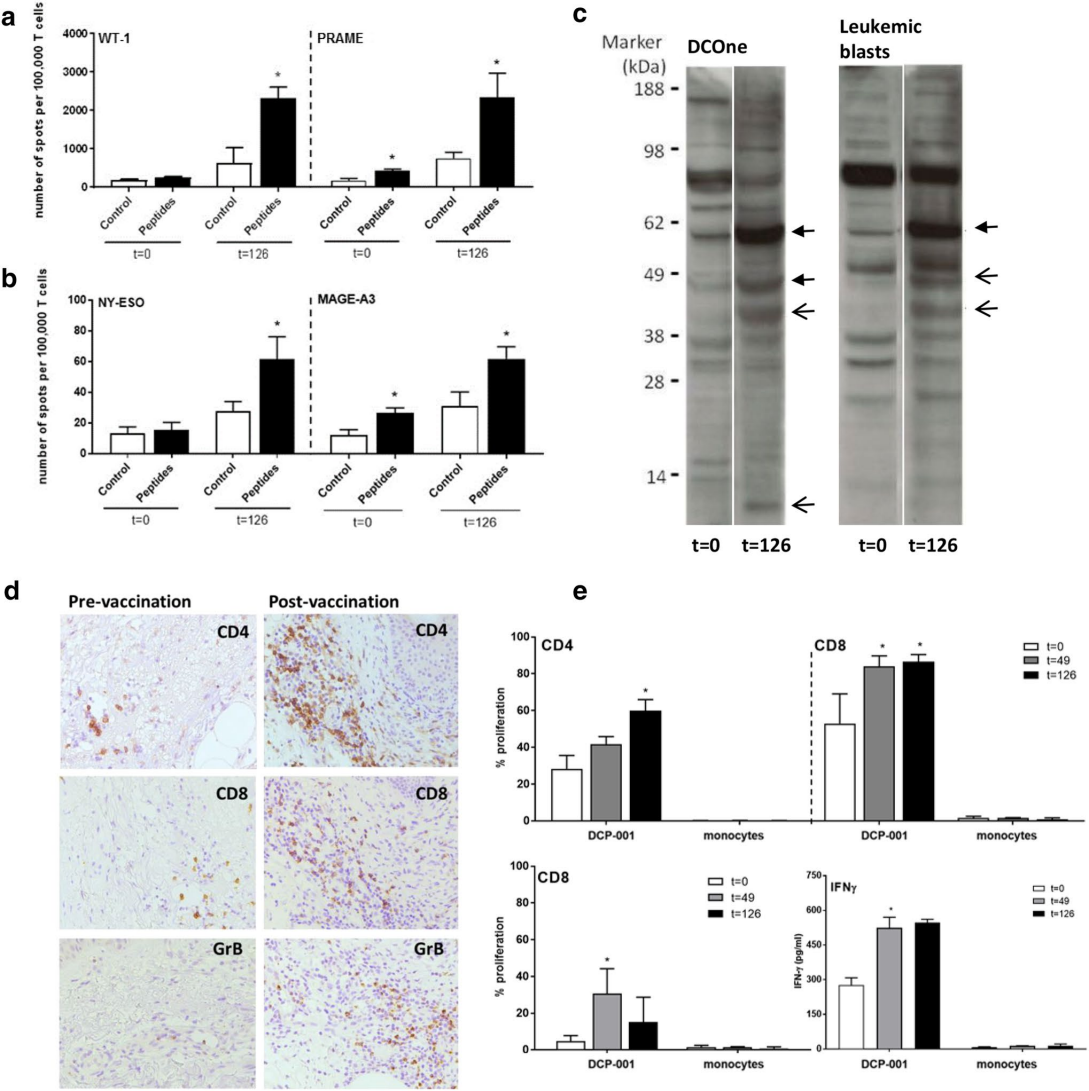
Immune responses induced by the DCP-001. Representative examples are shown of poly-functional immune responses elicited by DCP-001 vaccination. Pre- and post-vaccination T cell responses after in vitro restimulation in an IFNγ ELISpot read-out against **a** WT-1 and PRAME in patient 012 and against **b** NY-ESO-1 and MAGE-A3 in patient 015. **c** Examples of enhanced (closed arrows) and de novo serological responses post-vaccination against DCOne progenitor and autologous AML blast lysates in patient 006. **d** Examples of CD4, CD8, and Gr-B immunohistochemical staining of immune infiltrates in the dermis of DCP-001 DTH biopsies pre- and post-vaccination (magnification ×100). **e** Proliferation (by CFSE dilution read-out) of CD4^+^ or CD8^+^ T cells from peripheral blood (013), pre- (*t* = 0) and post-vaccination (*t* = 49 or 126 days), against DCP-001 mature DC (top panels), or their DCOne progenitors (bottom left panel) or IFNγ release in response to DCP-001 (right bottom panel); autologous monocytes served as non-tumor controls. *Gr-B* Granzyme B

T cell reactivity by IFNγ ELISpot analysis after in vitro stimulation was assessed against the AML-related antigens WT-1 and PRAME, both of which were expressed by the DCP-001, as well as against NY-ESO-1 and MAGE-A3. NY-ESO-1 and MAGE-A3 are not expressed by DCP-001 but were included to monitor for possible epitope spreading, i.e., T cell responses primed against epitopes released from the patients’ autologous blasts after a successful DCP-001 induced anti-AML immune response. Indeed, instances of induced de novo or boosted pre-existent responses were found post-vaccination for all four TAAs (Fig. 4a, b). Overall, four out of eight evaluable patients showed DCP-001 induced or enhanced WT-1, PRAME, MAGE-A3, or NY-ESO-1 responses (Table 3). An unexpectedly high number of patients (3 out of 3) showed enhanced post-vaccination responses against NY-ESO-1, of which one was de novo primed (Fig. 4b). For one patient (004), also pre-and post-treatment bone marrow samples were available revealing upregulated NY-ESO-1 T cell reactivity at day 126 (Supplementary Fig. 1). All tested patients showed pre-existent reactivity to the CEFT recall antigen pool, which was maintained over treatment, indicating good immune competence (data not shown). An overview of pre- and post-vaccination ELISpot responses of all four patients with post-vaccination-positive ELISpot reactivities (expressed as specific T cell numbers per 10^5^ T cells) are shown in Supplementary Fig. 2.

**Table 3:** Overview T cell immune-monitoring data

	Patient ID^a^	Δ T cell response to DCP-001^b^	TAA-specific T cell response WT-1^b^				Δ T cell influx at DTH site^b^	Δ DTH^b^	Overall T cell score^d^	Survival post-Vacc (months)
			WT-1	PRAME	NY-ESO-1	MAGE-A3				
Short survivors ≤ 6 months	002	ND	−	ND	ND	ND	−	−	0/3	6
	005	−	ND	ND	ND	ND	ND	+	1/2	2
	006	−	ND	ND	ND	ND	+	−	1/3	5
	012	+	+	+	ND	ND	+	−	¾	2
	014	ND	−	ND	ND	ND	−	−	0/3	3
Overall responses		1/3	1/3	1/1	0/0	0/0	2/4	1/5	5/15 (33%)	3.6
Long survivors > 6 months	001	−/+^c^	−/−^c^	−	ND	ND	+	+	2/4	36
	004	−	ND	+	+	ND	−	+	2/4	22
	007	−	−	−	ND	ND	+	−	1/4	63
	008	+	ND	ND	ND	ND	+	+	3/3	7^e^
	013	+	−	−	+	−	+	+	4/4	55
	015	+	−	+	+	+	ND	−	2/3	36
Overall responses		4/6	0/4	3/5	3/3	1/2	4/5	4/6	14/22 (68%)	36.5

*ID* identity, *ND* not done, *OS* overall survival

^a^Patient 11 (3 months OS) was excluded from this analysis; died from infection, no monitoring data available

^b^For definitions of positive responses: see “Materials and methods”

^c^s data derived after two additional booster vaccinations; T cell response to DCP-001 was negative after first round of vaccinations

^d^Based on positive T cell responses from evaluable datasets, *p* = 0.049 by two-sided Fisher’s exact test between ≤ 6 and > 6-month survival groups

^e^Patient 8 died because of infectious complications but was not progressive at time of death

Antibody responses generated against DCOne progenitor and autologous blast antigens was evaluated in 10/12 patients. Five patients showed an increased response with bands gaining in intensity or new bands appearing on the blots, denoting vaccination-induced humoral responses (Fig. 4c). Specificity of the responses, in terms of the identity of the recognized antigens, remains to be determined. Importantly, induced antibody responses against autologous blasts were also observed in two out of three evaluable patients (Fig. 4c), which demonstrates the induction of immunity against autologous AML blasts by the allogeneic DCP001.

#### DTH reactivity

In the DTH tests, 5 of 11 evaluable patients had an increase of ≥ 50% in the mean diameter of induration at day 51 compared to baseline, indicating DCP-001-induced T cell response (Table 3). Importantly, no reactivity to the Cryostor vehicle control was ever observed (data not shown). Of the 7 patients who did not have an increase at day 51 compared to baseline, all showed a baseline reaction of at least 4 mm (range 3.5–15 mm) which points to a pre-existing immunity to components of the DCP-001. This could be an allo-reaction but also a response to TAAs in the vaccine. Evidence for vaccination-induced increases in DTH reactivity was mostly found among long-term (> 6 months) survivors (Table 3).

#### Immunohistochemistry

Immunohistochemical assessment of DTH punch biopsies was performed for T cell and DC influx in 9 out of 12 patients at day 2 and day 51 (Fig. 4d). All of these patients showed an increased influx of activated T cells in response to the vaccine as compared to the Cryostor vehicle control in both the superficial and deep dermis and to a similar extent at d2 (pre-vaccination) and d51 (post-vaccination) (Supplementary Fig. 3). Irrespective of the dosage used, DCP-001 vaccination also resulted in increased TIA or Granzyme B-positive cytotoxic T cell infiltration in individual cases (Fig. 4d and Supplementary Fig. 3a). No CD56^+^ NK cell infiltration was observed. In addition, a selective increase of CD4^+^ and CD45RO^+^ infiltrate (in particular in the higher dose levels) in the superficial dermis was observed at the DCP-001 delivery site that may point to a more active effector-memory Th-cell compartment after vaccination (Supplementary Fig. 3b).

#### T cell reactivity to DCP-001 and its DCOne progenitors by MLR

The proliferative T cell capacity in response to DCP-001 and DCOne progenitor cells was analyzed in 9/12 patients. Seven of these patients showed pre-existent T cell responses (proliferation rate ≥ 20%), as is to be expected for an allogeneic vaccine. Increased proliferative responses (either CD4 or CD8) against DCP-001 cells and/or DCOne progenitor cells upon vaccination were observed in 6/9 evaluable patients (Table 3; Fig. 4e), whereas in none of the patients reactivity to autologous monocytes was observed. Notably, in five cases, increased post-vaccination reactivity to DCOne progenitors was observed, which almost exclusively involved CD8^+^ T cells (data not shown). This observation is highly suggestive for the induction of a CD8^+^ effector T cell response to AML blasts.

CBA analysis further showed a measurable IFNγ response against DCP-001 in 5/9 patients (for a representative result, see Fig. 4e), accompanied by IL-2 release in three cases. In two additional patients, also post-vaccination IL-4, IL-6, IL-17 or IL-10 responses were detected (Supplementary Fig. 5). These results demonstrate the induction of Th1 or mixed Th1/Th2/Th17 responses by DCP-001.

#### Immune reactivity induction upon a booster vaccination regimen

Patient 001 received two booster vaccinations of 10 million DCP-001 cells 13.5 months after initial treatment. Remarkably, whereas this patient did not show DCP-001-induced immune reactivity after the first four vaccinations, after receiving the booster vaccination she developed de novo antibody responses both to her autologous leukemic blasts and to DCOne progenitors (Supplementary Fig. 4) as well as proliferative CD8^+^ T cell responses against the DCP-001 (Table 3).

#### DCP-001-induced T cell reactivity in relation to survival

Overall, the immune-monitoring data clearly demonstrate DCP-001-induced T cell reactivity. When results from all T cell-related assays were combined, an “Overall T cell Score” was derived (Table 3). To arrive at the T cell score, vaccination-induced T cell immune responses were scored in four different categories, i.e., (1) T cell proliferative response to the DCP-001, (2) a positive post-vaccination leukemia-associated antigen-specific ELISpot reactivity, (3) increased number of infiltrating CD4^+^ or CD8^+^ T cells in the superficial dermis of the post-vaccination DTH site (by immunohistochemistry, see Supplementary Fig. 3b and 4) post-vaccination increase in DTH site induration. Positive responses in each of the categories (as defined in the “Materials and methods”) resulted in a point per category, with a low score of 0 out of 4 and a possible highest T cell score of 4 out of 4. These analyses demonstrated significantly lower scores (5 positive assays out of 15 conducted) in short-term survivors (≤ 6 months) versus long-term survivors. (> 6 months, 15 positive assays out of 22 conducted, *p* = 0.049 by two-sided Fisher’s exact test).

## Discussion

Persistent MRD after induction chemotherapy poses a major challenge in the treatment of AML. Particularly in older patients, who are not eligible for post-remission intensification treatments such as allogeneic hematopoietic stem cell transplantation, overall survival rates are dismal (15–20% at 2 years) [24–27]. Successful immunotherapy can offer long-term protection against outgrowth of MRD through the activity of memory T cells that can specifically recognize and eliminate leukemic blasts. That this may present an effective treatment option is supported by the apparent dependence of the efficacy of allogeneic hematopoietic stem cell transplantation on the graft–versus–leukemia effect, mediated by T cell immunity against histocompatibility-mismatched leukemic blasts [28]. Unfortunately, transplantation-associated graft–versus–host disease is associated with considerable morbidity and mortality in patients [29, 30]. In AML, DC-based vaccines have, therefore, been clinically explored as an alternative means of kick starting cell-mediated immunity against leukemia.

Previous clinically tested AML-targeted DC vaccines have mostly consisted of autologous monocyte-derived DC, either loaded with single-antigen-derived peptides or mRNA [7, 31–33] or autologous AML blast lysates [34, 35], fused to autologous AML blasts [36], or derived from autologous blasts that were differentiated into DC-like cells [37]. These approaches have led to varying levels of clinical efficacy with reports of dropping peripheral blast counts and prolonged overall survival in treated individual patients. Approaches employing autologous blasts rely on TAAs and neo-antigens selectively expressed in autologous tumors and provide a poly-epitope-based personalized vaccine that can trigger both Th, CTL and humoral immunity. A disadvantage of this personalized approach is the laborious and costly vaccine production process and its inherent variability in quality [9, 37]. As AML has a low mutational burden resulting in an expected low number of neo-epitopes [38], vaccination efficacy will most likely depend on shared common TAAs. As such a strong case can be made for an allogeneic DC-based approach that can offer a highly standardized off-the-shelf platform.

DCP-001 expresses a range of shared TAAs and HLA haplotypes that together cover more than 70% of the Caucasian population. It presents a highly potent, standardized and fully scalable vaccine platform that is widely applicable. Here, data of a phase-I trial of DCP-001 vaccination in elderly AML patients in CR1/CR2 or with smoldering disease are presented. The vaccine proved safe and a treatment regimen of four biweekly i.d. DCP-001 injections was feasible, thus meeting the primary objectives of the study. The obtained results further suggest that in patients with demonstrable immune competence and maintained peripheral T cell rates, DCP-001 can induce a multi-functional immune response to the vaccine, its AML progenitors, common shared leukemia-associated antigens (LAA), as well as to antigens in autologous leukemic blasts. Although the study size is too small to draw definitive conclusions, the observed immune responses may have translated into a long-term clinical benefit in those patients who were in CR at the time of vaccination, and therefore, would have been in a less immune-suppressed state. Typically, the inclusion criteria employed in this trial would select for patients with an average life expectancy of 3–6 months [1, 2, 39]. It is, therefore, remarkable that 6 of the 12 enrolled patients in this trial survived for more than 6 months after start of treatment (Fig. 2, median overall survival 36 months), with one patient still alive at the time of writing. Clearly, the small number of patients enrolled in this trial with as primary endpoint safety and feasibility does not allow for firm conclusions with regards to clinical outcome. As such, the results from this trial should be regarded as hypothesis generating. The hypothesis that DCP-001 induces immune responses against the patient’s leukemic blasts, translating into clinical benefit in immune competent patients who are in CR at the time of vaccination, will now be investigated in a multi-center randomized phase II trial in AML patients in CR1.

As a secondary objective, DCP-001-induced immune responses were assessed. Allogeneic DC vaccines have been shown to induce and support antitumor immunity in part through generation of allo-response-induced pro-inflammatory conditions conducive to Th1 skewing and in part through the direct priming of tumor-reactive or cross-reactive T cells [40, 41]. The observed induction of DTH responses against the vaccine and increased post-vaccination DTH reactivity in five of the tested patients are in line with the induction and boosting of systemic Th1 responses. Both CD4 and activated (based on a rise in Granzyme B and TIA expression) CD8 T cell responses were induced, as judged by immunohistochemistry analyses of biopsies taken from the DTH site. Results from in vitro proliferation assays with pre- and post-vaccination T cells in response to DCP-001 were in line with these observations. Vaccination-induced proliferative responses were noted upon in vitro restimulation in six out of nine patients, which may have included responses to both allogeneic and tumor-associated antigens. These DCP-001-induced T cell responses were marked by increased release of Th1 cytokines, either alone or combined with the release of Th2 and Th17 cytokines. The observation of CD8^+^ T cell proliferative responses to DCP-001 progenitors in five patients further indicated the induction of effector T cells able to respond to leukemic blasts lacking high levels of costimulatory molecules. Vaccination-induced or enhanced T cell responses to WT-1, PRAME, NY-ESO-1, and MAGE-A3 were observed in 4 out of 8 evaluable patients in this study. Whereas WT-1 and PRAME are expressed by DCP-001, NY-ESO-1 and MAGE-A3 are not. This suggests that T cell responses may have been both directly primed or re-stimulated by DCP-001 (or after cross-presentation of its contents), or indirectly through bystander activation and/or epitope spreading. Overall, these results demonstrate the development of cell-mediated immune responses to vaccination at all doses tested. Finally, also induction of humoral responses were observed to DCP-001, its progenitors and importantly, also to autologous leukemic blasts. The latter unequivocally demonstrates the induction of immunity against autologous AML blasts by the allogeneic DCP-001.

DCP-001-induced immune responses were observed in patients from all cohorts and not restricted to the higher dose levels. Overall T cell reactivity (expressed as ‘T cell score’, Table 3) was significantly higher in long-term survivors (> 6 months). Though more patients would be needed for conclusive evidence, this is suggestive of a causal relationship between DCP-001-induced antitumor immunity and clinical outcome. Of note, patients with a maintained low blast count were more likely to develop an immune response to the vaccine, advocating the testing of such immunotherapies in patients with less advanced stages of disease.

In conclusion, DCP-001 vaccination in elderly AML patients is safe, feasible and leads to the induction or boosting of multifunctional antitumor immunity. In patients with CR and stable peripheral T cell rate-prolonged overall survival was observed (median 36 months). These promising data warrant further testing of this allogeneic off-the-shelf DC vaccine in AML patients in post-chemotherapy complete remission either as monotherapy or in combination with hypomethylating agents or immune checkpoint inhibitors.

## Electronic supplementary material

Below is the link to the electronic supplementary material.

Supplementary material 1 (PDF 576 KB)

## Abbreviations

AML: Acute myeloid leukemia
CBA: Cytometric bead array
CR: Complete remission
CTL: Cytotoxic T lymphocyte
DC: Dendritic cell
DTH: Delayed-type hypersensitivity
GMP: Good manufacturing practice
HPS: Human pooled serum
HSCT: Hematopoietic stem cell transplantation
LAA: Leukemia-associated antigen
MLR: Mixed lymphocyte reaction
MRD: Measurable residual disease
PBL: Peripheral blood lymphocyte
QC: Quality control
SAE: Severe adverse event
TAA: Tumor-associated antigen
VU: Vrije Universiteit
