# Supplementary material for: A novel allogeneic off-the-shelf dendritic cell vaccine for post-remission treatment of elderly patients with acute myeloid leukemia
Source: Cancer Immunol Immunother. 2018 Jul 23;67(10):1505–18. doi: 10.1007/s00262-018-2198-9 (PMC6182404; doi:10.1007/s00262-018-2198-9)
Supplement: Supplementary file 1 — Supplementary material 1 (PDF 576 KB) [file 262_2018_2198_MOESM1_ESM.pdf]

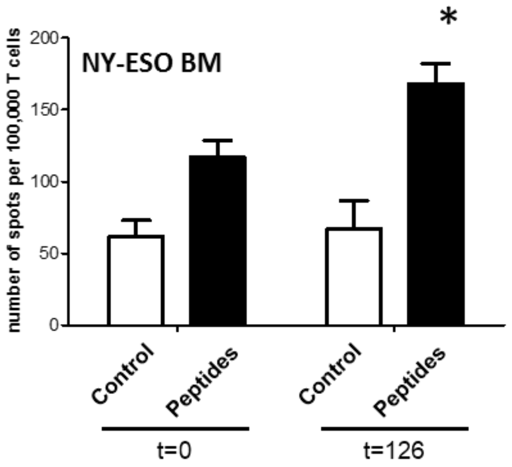

Supplementary Figure 1: DCP-001 induced T cell responses against NY-ESO-1 in bone marrow. Pre- (t=0) and post-vaccination (t=126) T cell responses after in vitro restimulation in an IFNγ elispot read-out against NY-ESO-1 overlapping 15-mer peptides in bone marrow samples of patient 004.

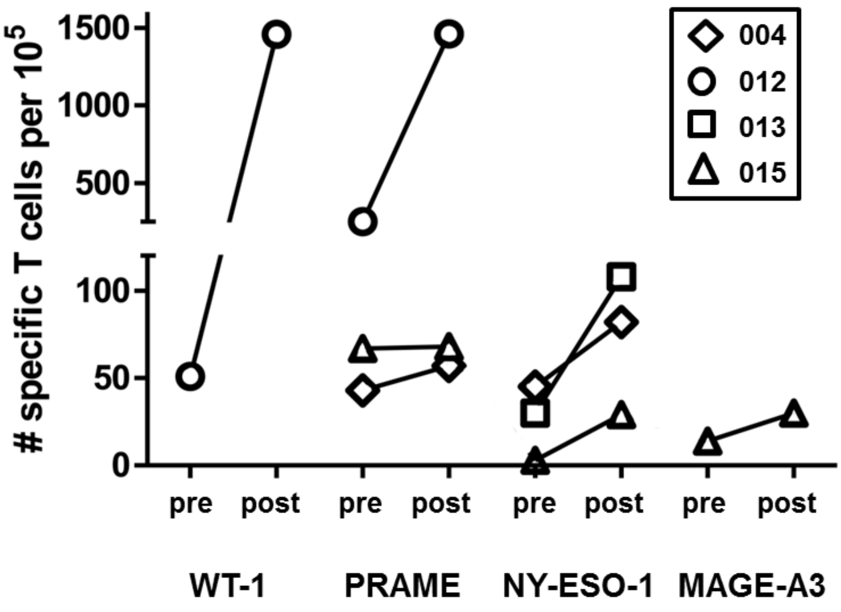

Supplementary Figure 2: Pre- and post-vaccination IFNγ ELIspot reactivity. Shown are ELIspot reactivities after in vitro stimulation (in number of antigen-specific T cells per 100,000, i.e. with vehicle background frequencies subtracted) for all ELIspot responses determined to be positive post-vaccination to the indicated tumor antigens. Definition of positive response: significantly higher than background controls in unpaired Student's T test, exceeding background at least 2-fold and with a minimum of 5 spots difference. The legend lists the corresponding patient numbers.

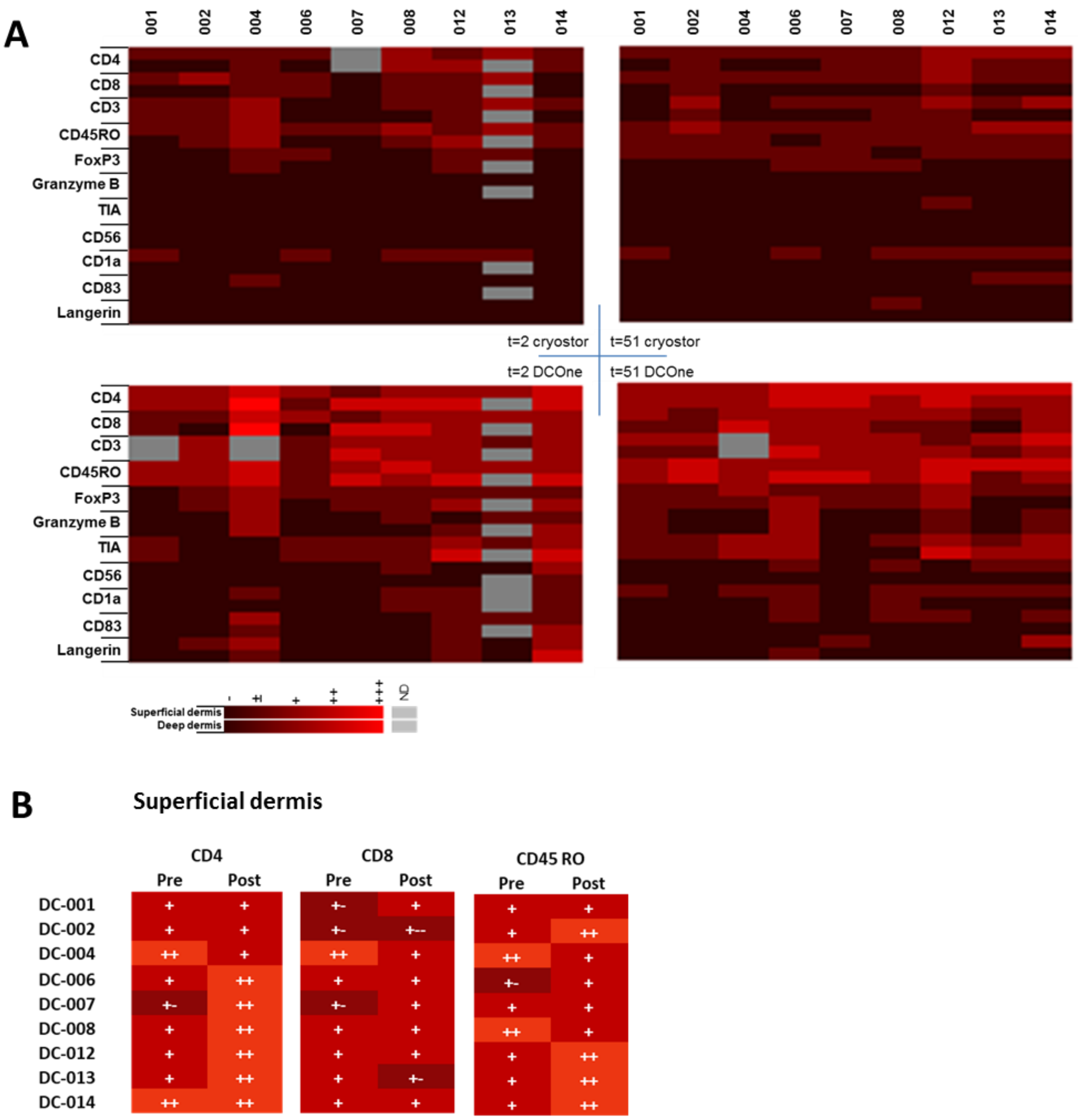

Supplementary Figure 3: Immune infiltrate analysis of pre- and post-vaccination delayed-type hypersensitivity (DTH) skin sites. Intradermal injections were administered of cryostor vehicle or DCP-001 cells at t=0 (pre-vaccination) and t=49 (post-vaccination). Two days later (t=2 and t=51) induration was measured and punch biopsies were taken from the DTH sites of the patients listed at the top (patients 001, 002, 004, 006, 007, 008, 012, 013, and 014). A) Immunohistochemical analysis (semi-quantitatively scored from – to +++) was performed for the markers indicated at the left. Relative expression levels in the superficical and deep dermis are indicated by intensity of the color red; grey=not done (ND). B) Grouped pre- and post-vaccination levels of infiltrating CD4⁺ and CD8⁺ T cells and CD45RO⁺ cells in the superfical dermis.

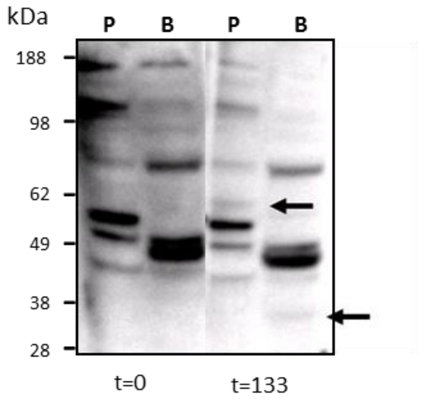

Supplementary Figure 4: Serological response induced upon 2 DCP-001 booster vaccinations. De novo induced antibody responses after two additional booster vaccinations with DCP-001 against lysates from DCOne progenitors (P) and autologous leukemic blasts (B) are indicated by arrows. Pre- (t=0) and post-booster (t=133) sera from patient 001 were used.

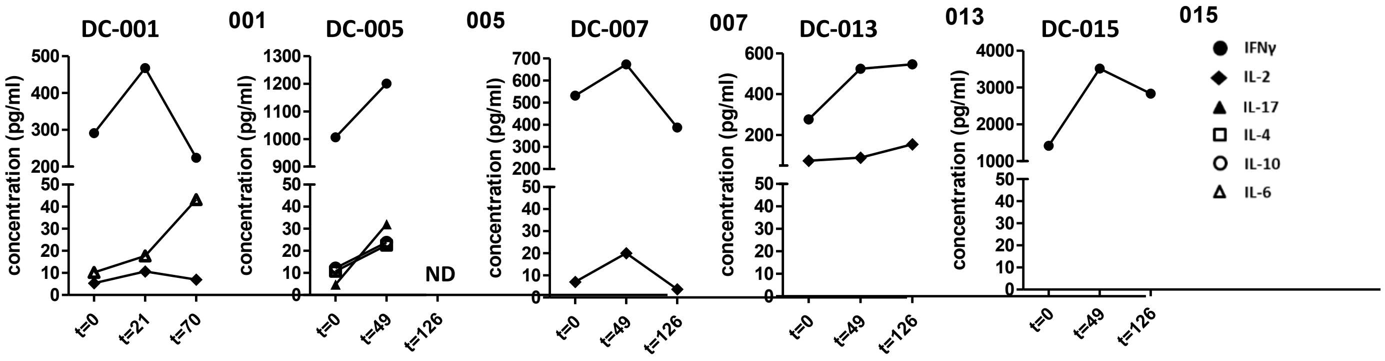

Supplementary Fig. 5 T cell cytokine responses to DCP-001 over the course of treatment. In vitro cytokine release by peripheral blood lymphocytes in response to DCP-001. Shown are results from patients with post-vaccination increased responses (significant increase from baseline; only positive responses shown).

Supplementary Table I. Prior (1st and 2nd line) therapy and cytogenetics of the enrolled patients

Patient ID	Lines of therapy prior to vaccination	Cytogenetics (most complex shown)
001	cytarabin, clofarabin, amsacrine, idarubicin,vidaza	46, XX [20]
002	daunomycin, cytarabin, tosedostat	46, XYt(1;3)(p36;q21)del(5)(q31,q33)[14]
004	daunomycin, cytarabin, bevacizumab, vidaza	46, XX [20]
005	daunomycin, cytarabin, aurorokinase inhibitor (AZD 1152)	nd
006	daunomycin, cytarabin, vidaza	46, XX t(2,2)(p21;p25)[4] /46 idem +1 der(1;21)(q10;q10)
007	daunomycin, cytarabine, lenalidomide	46, XX [20]
008	Idarubicin, ARA-c	46, XX [20]
011	Idarubicin, ARA-c, clofarabine AMSA	46, XX [4]
012	daunorubicine + cytarabine, vidaza	48, XY +21, +21 [9] /46,XY [11]
013	Idarubicin, cytorabine	45, XY [14] / 46,XY [6]
014	cytarabine, daunorubicine, Lenalidomide, vidaza	46, XY der(1;7)(q10;p10) [14] 46,XY [6]
015	Daunorubicin, cytarabin, lenalidomide	46,XY [20]

nd: not done
